# Supplementary material for: Phylogenetic and Functional Substrate Specificity for Endolithic Microbial Communities in Hyper-Arid Environments
Source: Front Microbiol. 2016 Mar 9;7:301. doi: 10.3389/fmicb.2016.00301 (PMC4784552; doi:10.3389/fmicb.2016.00301)
Supplement: Supplementary file 1 [file Presentation_1.PDF]

Supporting Information

Phylogenetic and Functional Substrate Specificity for Endolithic Microbial Communities from the Atacama Desert

Alexander Crits-Christoph, Courtney K. Robinson, Bing Ma, Jacques Ravel, Jacek Wierzchos, Carmen Ascaso, Octavio Artieda, Virginia Souza-Egipsy, M. Cristina Casero and Jocelyne DiRuggiero

- Table S1: Diversity metrics for the 16S rRNA gene sequences data
- Table S2: Functional abundance for SEED categories for NRP and PK
- Table S3. Functional assignment for SEED subsystems with significant differences between the two communities
- Fig. S1: Taxonomic composition at the phylum level using 16S rRNA gene sequences
- Fig. S2: Rarefaction curves of observed species (left) and phylogenetic diversity (right) using 16S RNA gene sequences
- Fig. S3: Taxonomic composition at the phylum level using 16S rRNA gene sequences assembled with EMIRGE
- Fig. S4: Distribution of KEGG super-pathways
- Fig. S5: Distribution of pathways involved in nitrogen assimilation
- Fig. S6: Phylogenetic tree of the cyanobacteria metagenomic bins.

Table S1: Diversity metrics for the 16S rRNA gene sequences data from the calcite and ignimbrite substrates with a 97% sequence similarity threshold (± standard deviation).

	OTUs observed richness	Shannon diversity index	Faith's phylogenetic diversity index	Chao1 diversity estimate	Pielou's evenness index
Calcite	272 ± 24	6.1 ± 0.1	15.9 ± 0.5	399 ± 48	0.75 ± 0.02
Ignimbrite	122 ± 32	4.3 ± 0.6	8.4 ± 1.3	197 ± 64	0.63 ± 0.06

Abbreviation: OTU, operational taxonomic units.
Analysis using data sets of equal size subsampled at 2400 sequence reads; average of duplicates.

Table S2: Functional abundance of all sequence reads assigned to SEED categories (MG-RAST) related to non-ribosomal peptides (NRP) and polyketides (PK) in the calcite and Ignimbrite metagenomes.

<u>All SEED categories related to NRP</u>

Level 1	Level 2	Level 3	Function	Calcite	Ignimbrite
Iron acquisition and metabolism	Siderophores	Siderophore assembly kit	Siderophore biosynthesis non-ribosomal peptide synthetase modules	8020	20440
Iron acquisition and metabolism	Siderophores	Siderophore pyochelin	Dihydroaeruginoate synthetase PchE, non-ribosomal peptide synthetase modules	97	46
Iron acquisition and metabolism	Siderophores	Siderophore pyochelin	Enantio-pyochelin synthetase PchF, non-ribosomal peptide synthetase module	114	249
**Total**				**8231**	**20735**
Iron acquisition and metabolism	Siderophores	Siderophore Pyoverdine	Non-ribosomal peptide synthetase modules, pyoverdine	3994	6293
Iron acquisition and metabolism	Siderophores	Siderophore Pyoverdine	Non-ribosomal peptide synthetase modules, pyoverdine??	1198	2232
Iron acquisition and metabolism	-	Iron acquisition in Vibrio	Non-ribosomal peptide synthetase modules, siderophore biosynthesis	0	106
Iron acquisition and metabolism	Siderophores	Siderophore Pyoverdine	Probable thioesterase involved in non-ribosomal peptide biosynthesis, PA2411 homolog	20	1
Regulation and Cell signaling	-	Pseudomonas quinolone signal PQS	Putative non-ribosomal peptide synthetase in AHQ biosynthetic operon	35	1
Iron acquisition and metabolism	Siderophores	Siderophore pyochelin	Pyochelin synthetase PchF, non-ribosomal peptide synthetase module	87	303
Iron acquisition and metabolism	Siderophores	Siderophore Pyoverdine	Pyoverdine sidechain non-ribosomal peptide synthetase PvdD	2020	3572
Iron acquisition and metabolism	Siderophores	Siderophore Pyoverdine	Pyoverdine sidechain non-ribosomal peptide synthetase PvdI	1160	2412
Iron acquisition and metabolism	Siderophores	Siderophore Pyoverdine	Pyoverdine sidechain non-ribosomal peptide synthetase PvdJ	1072	2077
**Total**				**9586**	**16997**
Secondary Metabolism	Bacterial cytostatics, differentiation factors and antibiotics	Nonribosomal peptide synthetases (NRPS) in Frankia sp. Ccl3	NRPS loading module Thr-PG-PG-Thr	196	173
Secondary Metabolism	Bacterial cytostatics, differentiation factors and antibiotics	Nonribosomal peptide synthetases (NRPS) in Frankia sp. Ccl3	NRPS module 2 PG-PG-Asn	43	46
Secondary Metabolism	Bacterial cytostatics, differentiation factors and antibiotics	Nonribosomal peptide synthetases (NRPS) in Frankia sp. Ccl3	NRPS module 3 PG-PG	56	17
Secondary Metabolism	Bacterial cytostatics, differentiation factors and antibiotics	Nonribosomal peptide synthetases (NRPS) in Frankia sp. Ccl3	NRPS module 4 PG-Ser-Gly-Thr	214	1638
**Total**				**509**	**1874**

All SEED categories related to PK

Iron acquisition and metabolism	Siderophores	Siderophore Yersiniabactin Biosynthesis	iron aquisition yersiniabactin synthesis enzyme (Irp1,polyketide synthetase)	1295	1796
Cell Wall and Capsule	Cell wall of Mycobacteria	mycolic acid synthesis	Phenolpthiocerol synthesis polyketide synthase ppsA	59	7
Cell Wall and Capsule	Cell wall of Mycobacteria	mycolic acid synthesis	Phenolpthiocerol synthesis polyketide synthase ppsB	154	302
Cell Wall and Capsule	Cell wall of Mycobacteria	mycolic acid synthesis	Phenolpthiocerol synthesis type-I polyketide synthase ppsC	344	1663
Cell Wall and Capsule	Cell wall of Mycobacteria	mycolic acid synthesis	Phenolpthiocerol synthesis type-I polyketide synthase ppsd	106	132
Cell Wall and Capsule	Cell wall of Mycobacteria	mycolic acid synthesis	Phenolpthiocerol synthesis type-I polyketide synthase ppsE	170	3
Dormancy and Sporulation	-	Spore pigment biosynthetic cluster in Actinomycetes	Polyketide beta-ketoacyl synthase WhiE-KS	290	1140
Dormancy and Sporulation	-	Spore pigment biosynthetic cluster in Actinomycetes	Polyketide beta-ketoacyl synthase WhiE-KS paralog	187	245
Dormancy and Sporulation	-	Spore pigment biosynthetic cluster in Actinomycetes	Polyketide chain length factor WhiE-CLF	163	332
Dormancy and Sporulation	-	Spore pigment biosynthetic cluster in Actinomycetes	Polyketide chain length factor WhiE-CLF paralog	142	94
Dormancy and Sporulation	-	Spore pigment biosynthetic cluster in Actinomycetes	Polyketide cyclase WhiE II	37	325
Dormancy and Sporulation	-	Spore pigment biosynthetic cluster in Actinomycetes	Polyketide cyclase WhiE VII	48	111
Miscellaneous	Plant-Prokaryote DOE project	At1g24340	Polyketide hydroxylase WhiE VII	234	90
Dormancy and Sporulation	-	Spore pigment biosynthetic cluster in Actinomycetes	Polyketide hydroxylase WhiE VIII	234	90
Clustering-based subsystems	-	CBSS-83332.1.peg.3803	Probable polyketide synthase, similar to many. e.g. gp|M63676|SERERYAA_1 S.erythraea first ORF of eryA gene, involved in complex polyketide formation in erythromycin biosynthesis.	24	2
**Total**				**2192**	**4536**

Table S3. Functional assignment of sequence reads to the SEED database for the calcite and ignimbrite communities. Number of sequence reads assigned to subsystems with significant differences between the two communities.

	Calcite	Ignimbrite
**Ectoine Biosynthesis**		
Aspartokinase (EC 2.7.2.4)	2	0
Ectoine hydroxylase (EC 1.17.-.-)	103	2
L-2,4-diaminobutyric acid acetyltransferase (EC 2.3.1.-)	62	4
L-ectoine synthase (EC 4.2.1.-)	130	2
Diaminobutyrate-pyruvate aminotransferase (EC 2.6.1.46)	2269	4218
**Betaine biosynthesis from glycine**		
Dimethylglycine N-methyltransferase	720	88
Glycine N-methyltransferase (EC 2.1.1.20)	841	21
Sarcosine N-methyltransferase	844	31
**Synthesis of osmoregulated periplasmic glucans**		
Beta-(1-->2)glucan export ATP-binding/permease NdvA (EC 3.6.3.42)	368	92
Cyclic beta-1,2-glucan synthase (EC 2.4.1.-) NdvB	944	88
Glucans biosynthesis glucosyltransferase H (EC 2.4.1.-) MdoH	363	89
Glucans biosynthesis protein C (EC 2.1.-.-)	21	2
Glucans biosynthesis protein D precursor	358	99
Glucans biosynthesis protein G precursor	176	26
OpgC protein	44	12
**Iron acquisition and metabolism**		
Ferric iron ABC transporter, ATP-binding protein	5346	1212
Ferric iron ABC transporter, iron-binding protein	1606	428
Ferric iron ABC transporter, permease protein	8436	984
Iron-uptake factor PiuC	1563	39
Periplasmic protein p19 involved in high-affinity Fe2+ transport	1	0
Putative high-affinity iron permease	145	16
Ferric hydroxamate ABC transporter (TC 3.A.1.14.3), ATP-binding protein FhuC	2786	2139
Ferric hydroxamate ABC transporter (TC 3.A.1.14.3), periplasmic substrate binding protein FhuD	645	7
Ferric hydroxamate ABC transporter (TC 3.A.1.14.3), permease component FhuB	1141	32
Ferric hydroxamate outer membrane receptor FhuA	8	0
Siderophore synthetase large component, acetyltransferase	182	0
Siderophore synthetase small component, acetyltransferase	333	31
Desferrioxamine E biosynthesis protein DesA	327	6
Desferrioxamine E biosynthesis protein DesB	355	2
Desferrioxamine E biosynthesis protein DesC	145	5
Desferrioxamine E biosynthesis protein DesD	426	16
Hypothetical protein associated with desferrioxamine E biosynthesis	6	0
Ferrichrome-iron receptor	14152	40875
**Membrane Transport**		
Manganese ABC transporter, ATP-binding protein SitB	9138	3670
Manganese ABC transporter, inner membrane permease protein SitC	2669	257
Manganese ABC transporter, inner membrane permease protein SitD	15134	11926
Manganese ABC transporter, periplasmic-binding protein SitA	6174	1462
Manganese transport protein MntH	6594	17737
**Nitrogen Metabolism (Denitrification)**		
Nitrous oxide reductase maturation periplasmic protein NosX	17	1
Nitrous oxide reductase maturation protein NosD	130	3
Nitrous oxide reductase maturation protein NosF (ATPase)	668	367
Nitrous oxide reductase maturation protein NosR	257	3
Nitrous oxide reductase outer-membrane lipoprotein NosL	25	0
Nitrous oxide reductase maturation transmembrane protein NosY	56	3
Nitrous-oxide reductase (EC 1.7.99.6)	214	0

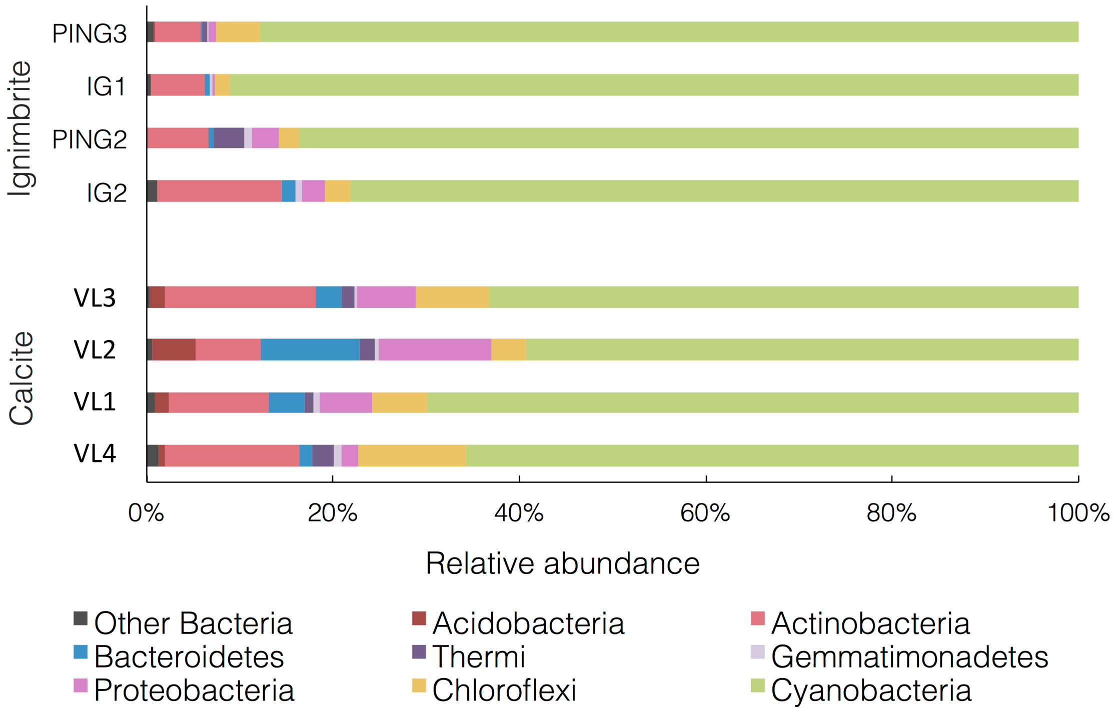

Fig. S1: Taxonomic composition at the phylum level of the calcite and ignimbrite communities using 16S rRNA gene sequences at a maximum sequencing depth of 2400 sequence reads. Phyla composing at least 1% of any sample were included. Each bar is the average of the data for 4 independent rocks. PING, IG, and VL are sample names for each rock (see Material and Methods for location).

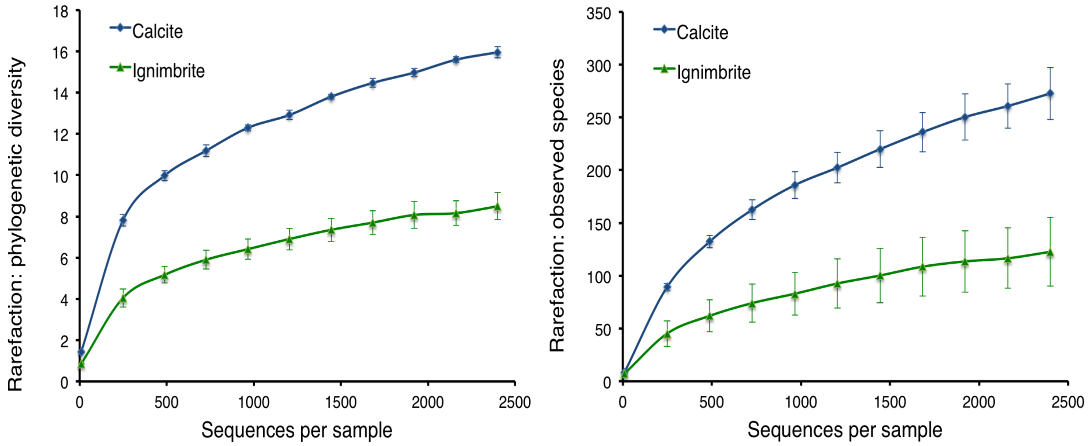

Fig. S2: Rarefaction curves of observed species (left) and phylogenetic diversity (right) for the calcite and ignimbrite communities using 16S RNA gene sequences at a maximum sequencing depth of 2400 sequence reads. Each curve is the average of the data for 4 independent rocks.

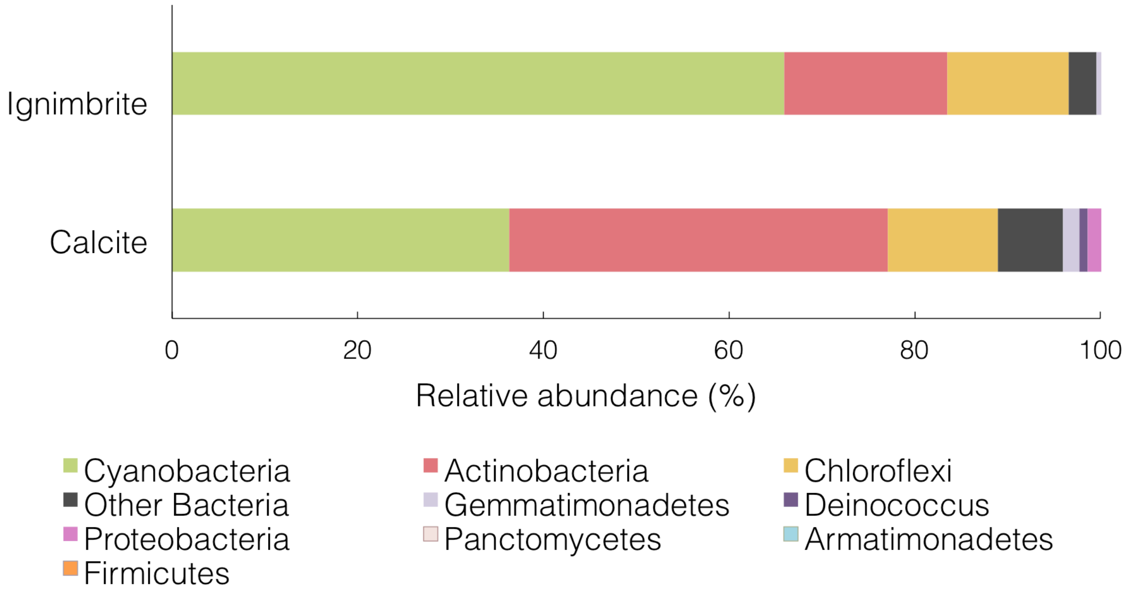

Fig. S3: Taxonomic composition at the phylum level of the calcite and ignimbrite communities using 16S rRNA gene sequences assembled with EMIRGE from the metagenome dataset.

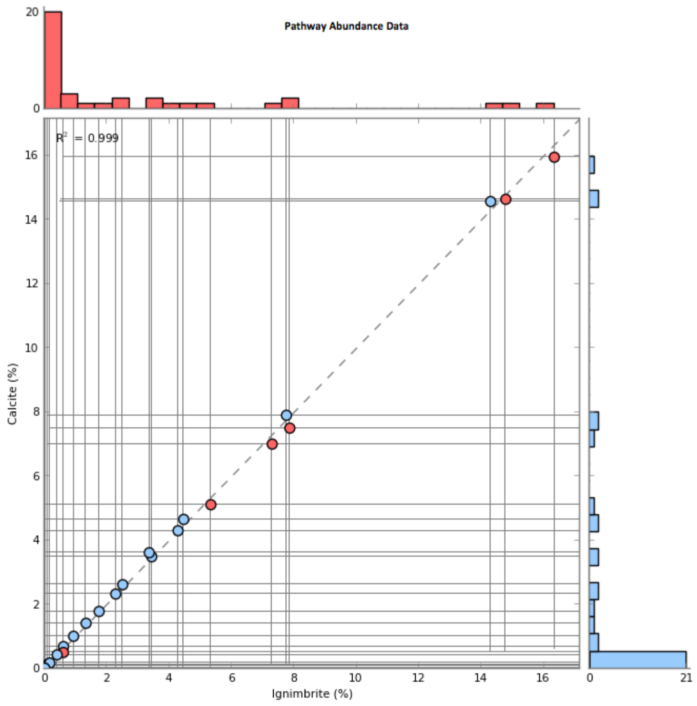

Fig. S4: Distribution of KEGG super-pathways between the calcite and ignimbrite communities using total sequence reads generated by STAMP.

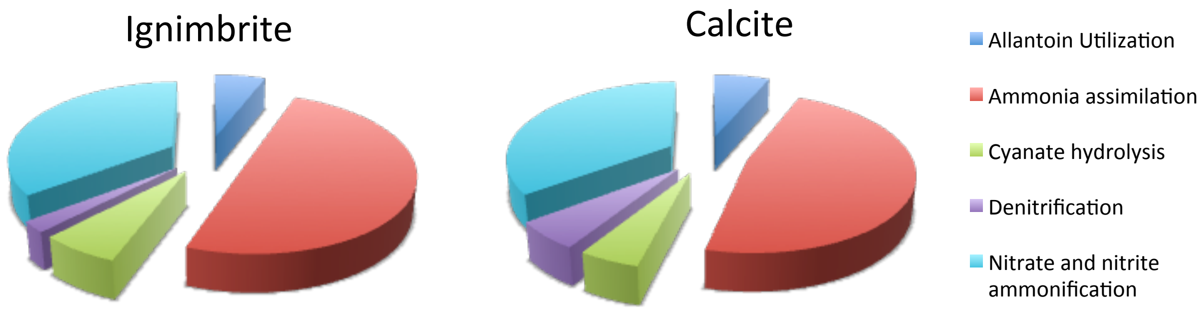

Fig. S5: Distribution of pathways involved in nitrogen assimilation for the calcite and ignimbrite communities using total sequence reads functionally annotated with SEED in MG-RAST.

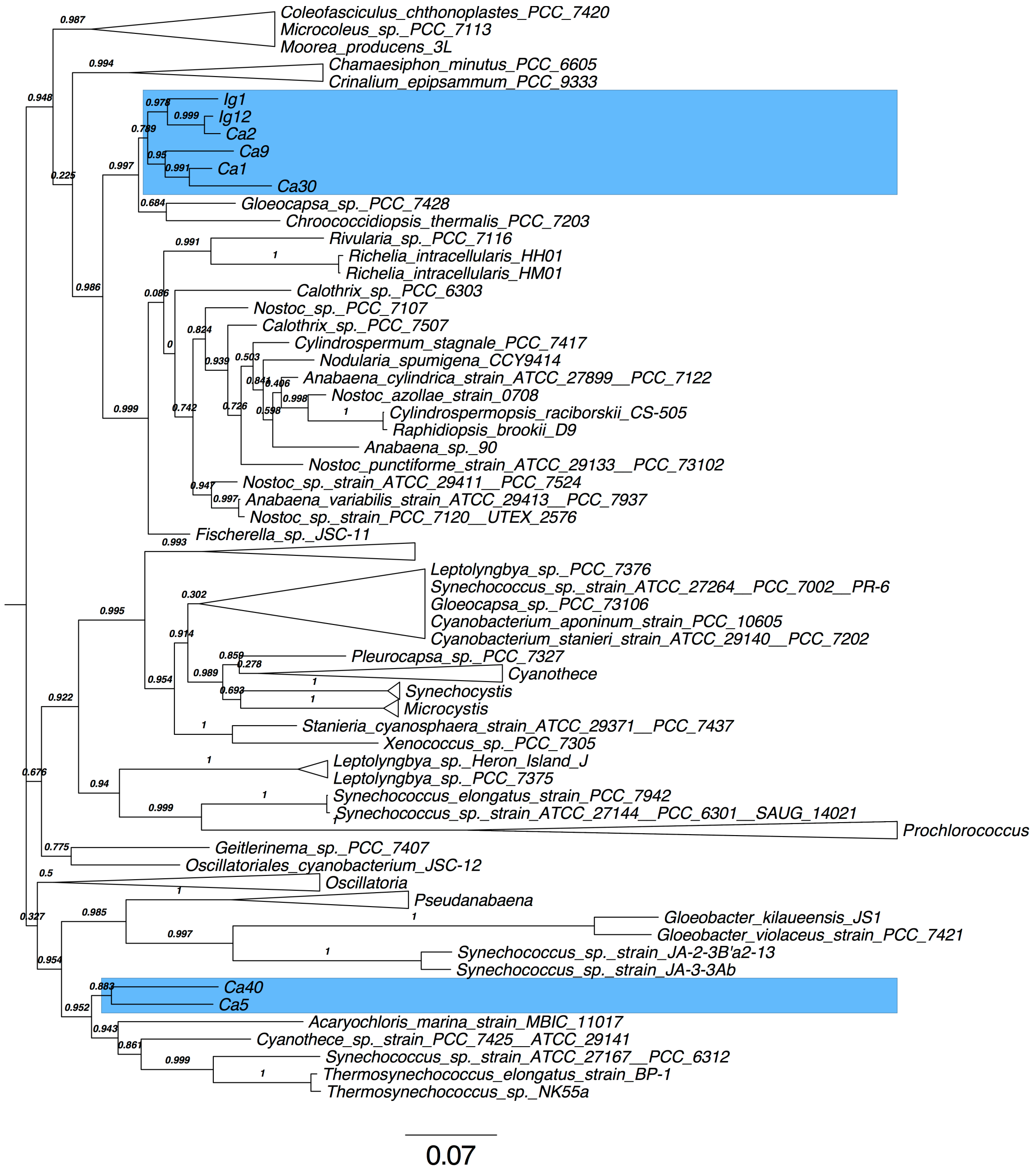

Fig. S6: Maximum likelihood phylogenetic tree of the cyanobacteria metagenomic bins from the calcite and ignimbrite communities built from a concatenation of 7 marker genes and compared with the same marker genes in all cyanobacteria references available in the UniProt database. The tree was generated using FastTree.
